# Ministernotomy approach to aortic arch inclusion and frozen elephant trunk in the treatment of acute Stanford A aortic dissection

**DOI:** 10.3389/fcvm.2022.944612

**Published:** 2022-09-07

**Authors:** Weitie Wang, Yong Wang, Hulin Piao, Zhicheng Zhu, Dan Li, Tiance Wang, Kexiang Liu

**Affiliations:** Department of Cardiovascular Surgery, The Second Hospital of Jilin University, Changchun, Jilin, China

**Keywords:** Stanford A aortic dissection, inclusion aortic arch procedure, novel aortic root repair, mild-moderate hypothermic technique, ministernotomy

## Abstract

This study aimed to report our results of ministernotomy approach to Liu’s aortic root repair technique, Liu’s aortic arch inclusion technique with frozen elephant trunk (FET) in the treatment in type A aortic dissection (TAAD). We retrospectively analyzed data on 68 Stanford A aortic dissection patients from October 2017 to March 2020. All patients underwent Liu’s aortic root repair technique, Liu’s aortic arch inclusion technique with FET and mild-moderate hypothermic circulatory arrest combined with ministernotomy approach. 154 TAAD patients between January 2014 and December 2016 underwent complete sternotomy were selected as control group. Clinical characteristics, data during operation, in-hospital and postoperative outcomes of these patients were observed. The mean hypothermic circulatory arrest time in ministernotomy Patients was 39.3 ± 7.9 min, aortic cross-clamp time was 105.9 ± 12.8 min, cardiopulmonary bypass time was 152.8 ± 24.3 min. Three patients died of multiple organ dysfunction syndrome in ministernotomy Patients. Perioperative temporary neurological dysfunction occurred in three (4.41%) patients, and 53 (77.9%) patients did not require any blood product transfusion during and after operation in ministernotomy Patients. Postoperative CT angiography (CTA) examination at 6-32 months showed excellent outcomes except in three (4.41%) cases where arch false lumen patency persisted. The Liu’s aortic root repair technique, Liu’s aortic arch inclusion technique with FET and mild-moderate hypothermia circulatory arrest simplify the surgical procedure and reduce bleeding, which can be accomplished through minimally invasive approach.

## Introduction

Type A aortic dissection (TAAD) is a life-threatening disease (1, 2). The surgical treatment includes ascending aorta replacement with or without hemiarch replacement. Since the false lumen of downstream poses a risk for reintervention, a more aggressive approach consisting of total arch replacement combined with frozen elephant trunk (TARF) in the descending aorta has been adopted and proven to be a safe and effective treatment (3–5). However, this surgical method is complex and uncontrollable bleeding, especially from the distal or posterior wall anastomosis, is the main cause of mortality (6). Additionally, all the above surgical procedures always combine deep hypothermia technique which destroy the coagulation mechanism and make hemostasis harder (7).

Thus, ministernotomy approach had not been widely used in the surgical treatment of Stanford A aortic dissection. In this study, Liu’s aortic root repair technique, Liu’s aortic arch inclusion technique with FET and mild-moderate hypothermia circulatory arrest simplify the surgical procedure and avoid serious complications, which can be accomplished through minimally invasive approach.

## Materials and methods

### Study population and design

This retrospective study was conducted by collecting data from 68 consecutive patients (39 men, mean age 52.11 ± 10.85 years) with TAAD who underwent surgery treatment from October 2017 to March 2020. All of the patients presented with serious chest or back pain and were diagnosed by computed tomography angiography (CTA). Among them, a history of hypertension was found in 47 patients (69.1%). Lower limbs malperfusion occurred in 2 patients (2.94%). All surgeries were completed in 14 days. 154 well matched patients baseline characteristics between January 2014 and December 2016 underwent complete sternotomy were selected as control group. The preoperative characteristics are shown in Table 1, which had no statistical difference between the two groups except the number of left subclavian artery involvement. This study was approved by the institutional ethics board of the Second Hospital of Jilin University (IRB:2017-032), China and a waiver for requirement of informed consent was obtained for all patients.

**TABLE 1:** Baseline and procedural characteristics.

	Ministernotomy patients (*n* = 68)	Full sternotomy patients (*n* = 154)	*P*-value
Age (years old)	52.1 ± 10.9	52.5 ± 11.4	0.807
Male	39 (57.4%)	93 (60.4%)	0.671
Acute (<14 day)	64 (94.1%)	144 (93.5%)	0.147
Marfan syndrome	1 (1.5%)	6 (3.8%)	0.340
Hypertension	47 (69.1%)	116 (75.3%)	0.335
Renal failure	3 (4.4%)	12 (7.7%)	0.355
Aortic regurgitation	8 (11.8%)	29 (18.8%)	0.193
Lower limb ischemia	2 (2.9%)	3 (1.9%)	0.646
Involvement of left subclavian artery	4 (5.9%)	1 (0.6%)	0.0154
Involvement of right subclavian artery	1 (1.5%)	1 (0.6%)	0.551

Ministernotomy surgical indications of TAAD was determined after screening for the following exclusion factors: (1) combination of other cardiac diseases, like mitral valve and ischemic heart disease, requiring intervention at the same time; (2) aortic dissection involving coronary artery; and (3) severe patients with unstable vital signs.

### Surgical method

The surgical techniques included Liu’s aortic root repair technique (8), Liu’s aortic arch inclusion technique with frozen elephant trunk (9), and mild-moderate hypothermic technique. The Liu’s aortic arch inclusion technique with frozen elephant trunk was indicated for all patients admitted for TAAD treatment after screening for the following exclusion factors: (1) a primary tear involving the orifices of the 3 brachiocephalic vessels; (2) a primary tear located between the innominate artery (IA) and left common carotid artery (LCCA) in the greater curve of the aortic arch.

### Surgical procedure

**FIGURE 1:**
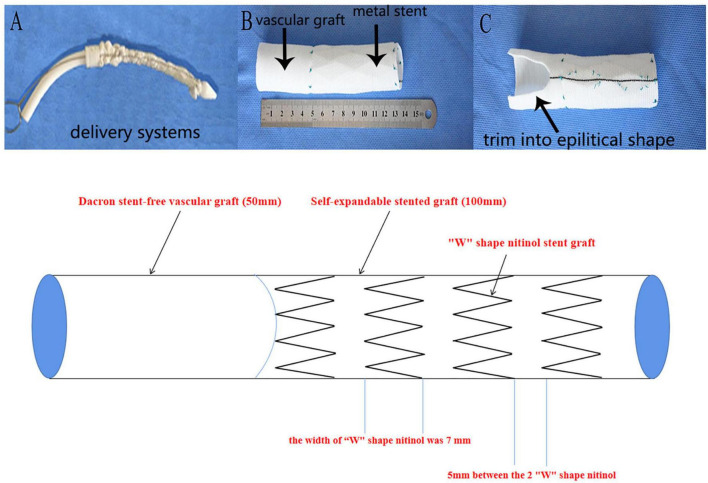
Self design stent graft. **(A)**: Compacted modified stent graft with the delivery system before implantation. **(B)**: Stent graft in expanded state: a distal 10 cm self-expandable nitinol stent graft and the proximal 50-mm dacron stent-free vascular graft. **(C)**: Example demonstration of the trimmed vascular graft used for aortic arch inclusion technique. **(D)**: Sketch of the self-designed stent graft: the stent graft was mainly composed of “W” shape nitinol material and dacron stent-free vascular graft.

Modification of the stent graft Cronus^®^ (Microport Medical Co., Ltd., Shanghai, China) consisted of a 10.0 cm self-expandable metallic SG and a 2.5–5 cm Dacron stent-free vascular graft at the proximal end which is enough to place inside the aortic arch for inclusion repair (Figure 1). The diameter of the distal aortic arch was measured by inserting a ball-shaped sizer to select the appropriate stent graft size. We used the Terumo (Japan or Inter Gard, France) one-branch vascular prosthesis for ascending aorta replacement (diameter, 24–30 mm).

**FIGURE 2:**
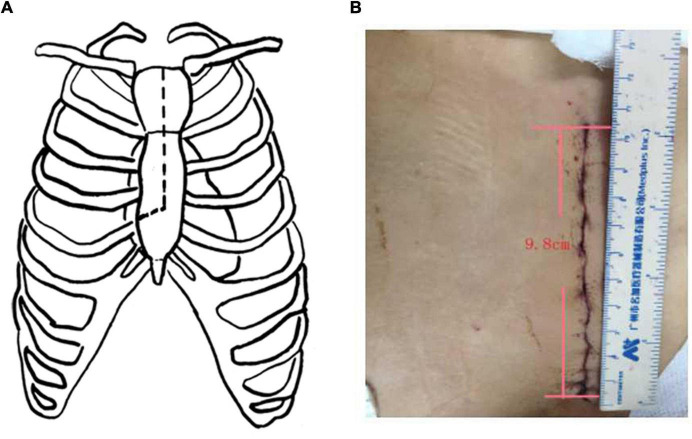
Upper J ministernotomy. **(A)**: The schematic diagram of upper J ministernotomy. **(B)**: Upper J ministernotomy after suture. The length of the incision was nearly 9 cm.

**FIGURE 3:**
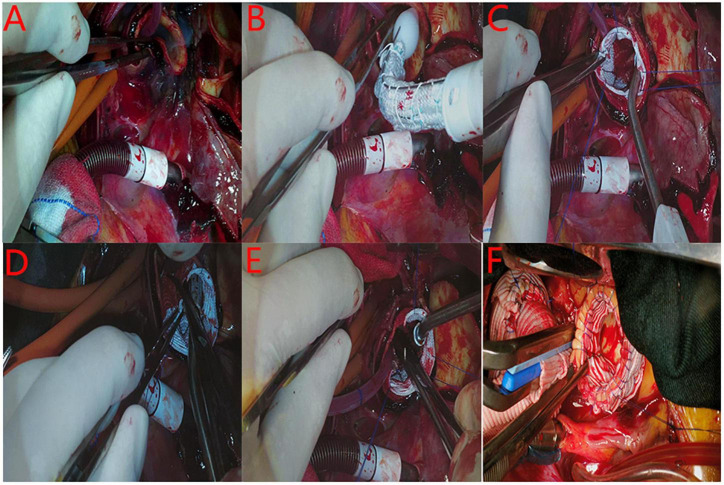
The procedure of the intra-aortic arch repair technique. **(A)**: Exposure of the posterior wall of the aortic arch for penetrate suturing. **(B)**: The frozen elephant trunk was inserted into the true lumen of the descending aorta. **(C)**: The stent-graft was released at 2 cm of the proximal of aortic arch. **(D)**: The stent-free vascular graft was trimmed into a wedge shape to expose the orifices of three brachiocephalic vessels. **E**: The trimmed vascular graft was penetrate sutured with the aortic arch. **(E)**: The remodeling of proximal aortic arch stump was finished. **(F)**: End-to-end anastomosis between the proximal aortic arch and the one-branch vascular graft is performed.

A median ministernotomy was performed from the superior sternal fossa, extending downward about 9–13 cm by a conventional vertical saw to transect the upper portion of the sternum from the sternal notch vertically to the 4th right intercostal space (upper J ministernotomy) (Figure 2). A cardiopulmonary bypass (CPB) was established via cannulation of the right axillary artery, superior vena cava, femoral artery, and vein. The left side of the heart was vented through the right superior pulmonary vein. Three brachiocephalic arteries, cross-clamped ascending aorta at the proximal of innominate artery, and myocardial protection were exposed with antegrade infusion of histidine-tryptophan-ketoglutarate (HTK) solution directly through coronary ostium. Circulatory arrest was started at a rectal temperature of 32°C; selective cerebral perfusion (SCP) was started via the right axillary artery. Transection of the ascending aorta at the proximal innominate artery and bilateral antegrade cerebral perfusion was accomplished through the left common carotid artery cannula. The flow was 600-800 mL min^–1^ under monitoring of regional cerebral oxygen saturation. After exposure of the posterior wall of the aortic arch (Figure 3A) a frozen elephant trunk (FET) of appropriate size was chosen and inserted into the true lumen of the descending aorta (Figure 3B). The stent-graft was released at 2 cm of the proximal of aortic arch (Figure 3C) and the vascular graft was trimmed into an elliptical shape to expose the orifices of three brachiocephalic vessels (Figure 3D). The trimmed vascular graft was sutured with aortic arch. The sutures started from the outside to inside and back to outside, through all layers of aortic arch wall and vascular graft (Figure 3E). After repairing the aortic arch, another two 5-mm vascular strips were sutured to the inside and outside of the aortic arch wall to strengthen the remaining 1 quarter of the proximal aortic arch stump. Then, end-to-end anastomosis with 4-0 polypropylene suture between the proximal aortic arch and a one-branch vascular graft was performed (Figure 3F; 9). Afterward, cerebral perfusion was discontinued, and systemic circulation was gradually restarted. Rewarming and aortic root reinforcement began as described below.

The aortic root was reinforced by placing vascular graft rings inside and outside the aortic root. A vascular graft of appropriate size was chosen (Vascutek Limited, United Kingdom), from which we cut three 1.5–2 cm wide vascular rings. One of the vascular rings was placed inside the aortic root and attached to the intima of the aortic wall, situated above the aortic valve commissures. The other two vascular rings were split longitudinally and placed on the outside surface of the aortic root wall. Three 4-0 polypropylene mattress sutures were placed at the aortic valve commissures for valve resuspension and served as the starting points for a running horizontal mattress suture to achieve an initial reinforcement of all three layers of the proximal ascending aorta: the inner vascular graft, the native aortic wall, and the outer vascular graft. This suture running along a horizontal line was situated just above the level of the three aortic valve commissures and coronary artery orifices, to close the false lumen from the beginning of the ascending aorta. The other three layers were reinforced by a continuous running 4-0 polypropylene suture placed at the distal end of the aortic root. Then, 3 to 5 additional 4-0 polypropylene vertical mattress sutures were placed above between the proximal and distal horizontal sutures within the proximal reconstructed aortic root, to further split the false lumen into pieces and promote the process of false lumen thrombosis. Thus, the aortic root reinforcement procedure was completed (8).

Finally, the proximal ascending aorta was anastomosed with a one-branch vascular graft. If the patients with primary entry locating in aortic arch between the left common carotid artery (LCAA) and LSA or the subclavian artery was involved, proximal of LSA was ligation and FET was released below LCAA. The branch of Terumo (Japan or Inter Gard, France) one-branch vascular prosthesis was used to ascending aorta-left subclavian artery bypass. Patients were gradually weaned off CPB. The remainder of the surgical procedure was performed as per routine practice, including hemostasis and closure of incisions. Video had been uploaded as Supplementary material.

### Data collection

All the data were collected from our local databases by cardiac surgeons, intensive care unit (ICU) personnel, nurse, anesthesiologists, and perfusionists. The status of the false lumen on imaging was classified as patent if flow was present in the absence of thrombus, as partially thrombosed if both flow and thrombus were present, or as completely thrombosed if no flow was present.

### Statistical analysis

Continuous data were expressed as a mean ± standard deviation and were compared using the t test, categorical variables were expressed as numbers (percentages). All statistical analyses were conducted by SPSS 20.0 on the computer.

## Results

Intraoperative data are presented in Table 2. The upper J ministernotomy was performed by the same surgeon in all patients who underwent the surgical treatment. In ministernotomy group, 4 patients with primary entry locating in aortic arch, the subclavian artery was involved and they underwent ascending aorta-left subclavian artery bypass. In ministernotomy group, 1 patient right subclavian artery was involved, who underwent ascending aorta-right subclavian artery bypass. Lower limb ischemia occurred in 2 patients in ministernotomy group, they underwent ascending aorta-left/right femoral artery bypass.

**TABLE 2:** Intraoperative data.

	Ministernotomy patients (*n* = 68)	Full sternotomy patients (*n* = 154)	*P*-value
Circulatory arrest time (min)	39.3 ± 7.9	38.1 ± 6.7	0.246
Aortic cross-clamp time (min)	105.9 ± 12.8	95.3 ± 10.2	<0.0001
CPB time (min)	152.8 ± 24.3	137.2 ± 26.2	<0.0001
Temperature	29.6 ± 0.38 °C	27.3 ± 1.24°C	<0.0001
Ascending aorta-left subclavian artery bypass	4 (5.9%)	1 (0.6%)	0.015
Ascending aorta-right subclavian artery bypass	1 (1.5%)	1 (0.6%)	0.551
Ascending aorta-left femoral artery	1 (1.5%)	1 (0.6%)	0.551
Ascending aorta-right femoral artery	1 (1.5%)	1 (0.6%)	0.551

All the operations were successfully completed with no intra-operative death. In ministernotomy group, none of the patients required conversion to full sternotomy. The mean hypothermic circulatory arrest temperature in ministernotomy patients was 29.6 ± 0.38°C, which is significant higher than 27.3 ± 1.24°C in full sternotomy patients (*P* < 0.05). The mean hypothermic circulatory arrest time in ministernotomy patients and full sternotomy patients was 39.3 ± 7.9 min and 38.1 ± 6.7 min, respectively (*P*>0.05). The aortic cross-clamp time and CPB time in ministernotomy patients were105.9 ± 12.8 min and 152.8 ± 24.3 min, which is significantly longer than 95.3 ± 10.2 min and 137.2 ± 26.2 min in full sternotomy patients (*P* < 0.05).

In ministernotomy group, three patients (4.41%) died of multiple organ failure after operation at 7 days, 8 days, and 11 days after operation. 8 patients died in full sternotomy group, which had no significant difference with ministernotomy group (*P*>0.05).

5 patients and 12 patients underwent continuous renal replacement therapy (CRRT) in ministernotomy group and full sternotomy group, respectively. Additionally, temporary neurological dysfunction occurred in 3 patients in ministernotomy group, but they recovered without complications before discharge. Postoperative outcomes are shown in Table 3. Surgical re-exploration for bleeding was not found in ministernotomy patients. The mean ICU stay was 4.9 ± 6.9 days, the mean hospital stay was 16.8 ± 6.9 days in ministernotomy group, which had no significant difference with those in full sternotomy group (*P*>0.05). 53 (77.9%) patients did not need any blood products transfusion during and after operation in ministernotomy group, which had significant difference with that in full sternotomy group (*P* < 0.05). There were no early events, such as paraplegia, cerebral infarction, and limb ischemia.

**TABLE 3:** Postoperative outcomes.

	Ministernotomy patients (*n* = 68)	Full sternotomy patients (*n* = 154)	*P*-value
ICU stay (day)	4.9 ± 6.9	4.6 ± 8.2	0.827
Hospital stay (day)	16.8 ± 6.9	18.9 ± 8.2	0.067
Low output syndrome	0	2 (1.3%)	0.345
Re-sternotomy for bleeding	0	2 (1.3%)	0.345
Renal dysfunction requiring dialysis	5 (7.4%)	12 (7.8%)	0.910
Neurological events	3 (4.4%)	14 (9.1%)	0.227
Pericardial tamponade	0	1 (0.6%)	0.511
DSWI	0	1 (0.6%)	0.511
No blood transfusion	53 (77.9%)	39 (25.3%)	<0.0001
30-days mortality 0%	3 (4.4%)	8 (5.2%)	0.804
False lumen patency persisted	3 (4.4%)	13 (11.1%)	0.285

**FIGURE 4:**
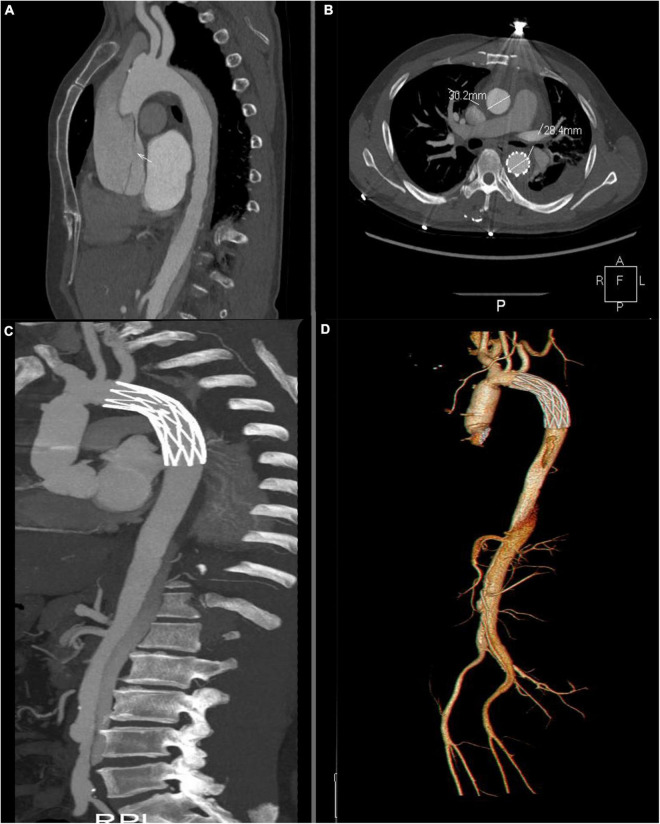
Computed tomography angiography assessment. **(A)** Intimal tear in the ascending aortic before the operation. The white arrow indicates the intimal tear. **(B)** Postoperative examination at 12 month. Thrombosis of the thoracic false lumen in descending aortic. **(C,D)** Postoperative volume-rendered image.

ICU = Intensive Care Unit; DSWI = Deep Sternal Wound Infection;

All of the patients underwent CTA examination in 6-32 months. False lumen closure rate of the descending aorta in ministernotomy group was found in 57 (83.8%) patients, while false lumen patency persisted in 3 (4.41%) patients and partial closure in 4 (5.88%). Complete aortic arch false lumen closure was achieved in 60 patients (88.2%) in ministernotomy group, which is significantly improved than 77.8% in full sternotomy group (P<0.05). Arch false lumen patency persisted in 3 (4.41%) patients and partial closure in 2 (2.94%) (Table 3 and Figure 4). Patients’ follow up CTA examination with false lumen patency persisted reported slightly enlargement comparing with preoperation. They were asymptomatic. Therefore, we continued to monitor these patients carefully (Table 4).

**TABLE 4:** Details of the aorta of patients with patent false lumen of aortic arch.

Patients (*n* = 5)	Preoperatively	At discharge	Postoperatively follow up
Arch	34.1 ± 4.3	35.8 ± 5.9	36.9 ± 5.1
True lumen of Arch	28.1 ± 4.8	27.5 ± 5.3	26.9 ± 5.2
False lumen of Arch	5.9 ± 4.3	8.3 ± 5.5	9.9 ± 4.9
Descending thoracic aorta	35.2 ± 3.1	36.5 ± 3.7	38.9 ± 5.6
True lumen of descending thoracic aorta	21.5 ± 3.6	21.9 ± 3.9	22.9 ± 4.9
False lumen of descending thoracic aorta	14.3 ± 2.8	14.6 ± 3.9	15.9 ± 5.5

Median follow-up time was 11.2 ± 9.2 months (6.0 - 32.0 months) in 65 patients. No patient was lost to follow-up, and all patients survival within the follow-up period.

## Discussion

The most common treatment for TAAD is ascending aorta replacement, with or without hemiarch replacement, in which the false lumen of the downstream aorta poses a risk for reintervention. Thus, TARF has been adopted as the main treatment for TAAD (10). However, this surgical method is complex and sometimes uncontrolled bleeding, especially from the distal aortic arch anastomosis site, is the main cause of mortality (11). Minimally invasive approaches were not used to treat TAAD to date. Our surgical treatment simplified the complex procedure and avoided uncontrolled bleeding. Mild-medium hypothermia reduces the destruction of coagulation mechanism and prevents errhysis during operation. Therefore, our surgical treatment made the ministernotomy approach possible in TAAD.

In Liu’s aortic arch inclusion technique with frozen elephant trunk (9), we do not need to extensively disassociate or resect the aortic arch because the whole arch procedure was performed inside the aortic arch. The three brachiocephalic vessels did not need to be replaced, which would reduce the number of anastomosis sites. The distal end-to-end anastomosis of aortic arch was also avoided in our technique, so bleeding from the distal anastomosis site of aortic arch could be avoided. In this time, we modified our inclusion aortic arch procedure through exposure of the posterior wall of the aortic arch and sutures through all layers of the aortic arch posterior wall and vascular graft. These operation slightly increase the circulatory arrest time, aortic cross-clamp time and CPB time than our previous method (9). Additionally, the stent graft covered the LSA if the intimal entry was near the LSA, we ligated the proximal LSA and inserted the bypass from the ascending aortic artery to LSA. However, combining these two methods, the early aortic arch false lumen closure rate approximately improved from 77.8 (9) to 88.9%. In addition, the increasing circulatory arrest time, aortic cross-clamp time and CPB time did not add the risk of postoperative complications comparing with complete sternotomy group.

Traditional strategies used for reinforcing the aortic root include usage of a felt strip, adventitial inversion technique, and neomedia technique (12). However, the chances of aortic root bleeding and false lumen patency persistence in the aortic root could not be eliminated (13). Surgical glue is another technique used in root reinforcement, but this technique was associated with a high incidence of intima necrosis and false aneurysm at the anastomosis and embolization (14). False aneurysm and pressure in the false lumen compress the coronary artery causing low cardiac output syndrome after operation. Liu’s aortic root repair technique consisted of placing the vascular graft ring inside and outside the aortic root, and the false lumen from the beginning of the ascending aorta was closed. Moreover, the false lumen was divided into pieces by 3–5 vertical mattress suture for reducing pressure, which would promote thrombosis. In the meantime, with vascular graft ring placed inside and outside the aortic root, totally avoiding new intima tear during anastomosis, which would avoid uncontrollable bleeding. Thus, the hemorrhage and anastomosis site pseudo-aneurysm were effectively avoided by our aortic root repair technique. During the early term follow-up, new intima tear was not found in all cases and low cardiac output syndrome was also avoided by this novel technique.

Deep hypothermic circulatory arrest (DHCA) has been widely used for surgical repair of aortic arch dissection (15). The advantage of this technique is good for organs and cerebral protection. However, the risk of coagulopathic bleeding and blood product transfusion, including coagulation factor consumption, caused by prolonged periods of CPB support and hypothermia-related platelet dysfunction are the main disadvantages of this technique (16). Prolonged CPB time, including longer cooling and rewarming time, caused by deep hypothermic technique may induce dysfunction of hemostasis, resulting in pronounced bleeding. Moreover, the effect of profound hypothermia on platelet function is also leads to bleeding. To avoid disadvantages of deep hypothermic technique, mild-moderate hypothermic technique has been used in our center since 2014 and proved to have many advantages, such as reducing the time of CPB and destruction of coagulation factors which make hemostasis easier. Furthermore, we used bilateral antegrade cerebral perfusion and near-infrared spectroscopy for real-time monitoring of the oxygen content of cerebral tissue to monitor brain protection; permanent neurological deficit did not occur in our study. The cooling blanket was applied to the back of the patient for local cooling after induction of general anesthesia to protect the spinal cord, and no paraplegia occurred in all cases. Therefore, we believe that the mild-moderate hypothermic technique is safe with adequate perfusion flow during SCP and local hypothermia of spinal cord.

Different minimally invasive approaches such as partial upper/lower sternotomy, right parasternal thoracotomy or transverse sternotomy, a lower half ministernotomy (T incision),V-shaped and Z-shaped partial ministernotomy to reduce surgical trauma have been used in cardiac surgery for nearly two decades. However, the upper partial sternotomy with J-shaped seem to more suitable for the operation of TAAD. Actually, ministernotomy could reduce bleeding from the sternal border. But the drawbacks of physically limited exposure and little space to operate were also disturbing. Liu’s aortic root repair technique, Liu’s aortic arch inclusion technique with FET and mild-moderate hypothermia circulatory arrest simplify the surgical procedure and avoid serious complications, which make ministernotomy possible during TAAD. Combined the simplify the surgical procedure with minimally invasive approach, no blood product transfusion requirement rate increased from 25.3% to 77.9% in ministernotomy group.

In this study, all the patients were suggested to reexamination at 6 months after discharge. The survival rate obtained by phone and e-mail follow-up way. In addition, there were 5 patients with arch false lumen patency persisted or partial closure before discharge. CTA reexamination at 6 months showed that diameter of true lumen of arch was decreased and false lumen was increased with no significant comparing with the diameter before discharge. They were asymptomatic and were dealt with by annual reexamination.

## Limitations

The current trial has several limitations. First, this study was a retrospective observational study in a single center which may influence the generalizability. A final validation would need a prospective, multi-center study with a larger sample size. Second, cases in this study were few and there was a lack of a longer follow-up period; hence, more cases with long-term follow-up should be reported in the future.

## Conclusion

Liu’s aortic root repair technique, Liu’s aortic arch inclusion technique with FET simplify the surgical procedure and eliminated surgical bleeding during operation, which can be accomplished through minimally invasive approach.

## Ethics statement

The studies involving human participants were reviewed and approved by the Institutional Ethics Board of the Second Hospital of Jilin University (IRB:2017-032), China. Written informed consent was not required for this study, in accordance with the local legislation and institutional requirements.

## Author contributions

KL and WW designed and wrote the study. YW and HP performed the analysis work. TW, DL, and ZZ organized the manuscript. KL and WW supervised the study. All authors reviewed the final manuscript.

## Abbreviations

TAAD: type A aortic dissection
CTA: computed tomography angiography
CPB: cardiopulmonary bypass
HTK: histidine-tryptophan-ketoglutarate
SCP: selective cerebral perfusion
FET: frozen elephant trunk
LSA: left subclavian artery
ICU: intensive care unit
CRRT: continuous renal replacement therapy
DHCA: deep hypothermic circulatory arrest.
